# Influence of multiple *APOE* genetic variants on cognitive function in a cohort of older men – results from the Normative Aging Study

**DOI:** 10.1186/s12888-014-0223-x

**Published:** 2014-08-01

**Authors:** Diddier Prada, Elena Colicino, Melinda C Power, David G Cox, Marc G Weisskopf, Lifang Hou, Avron Spiro III, Pantel Vokonas, Jia Zhong, Marco Sanchez-Guerra, Luis A Herrera, Joel Schwartz, Andrea A Baccarelli

**Affiliations:** Department of Environmental Health, Harvard School of Public Health, 665 Huntington Ave, Boston, MA 02115 USA; Department of Epidemiology, Harvard School of Public Health, 665 Huntington Ave, Boston, MA 02115 USA; Department of Epidemiology, Johns Hopkins Bloomberg School of Public Health, 615 N, Wolfe Street, Baltimore, MD 21205 USA; INSERM U1052, Centre de Recherche en Cancérologie de Lyon, Lyon, F-69000 France; Centre Léon Bérard, Pole de Recherche Translationnelle, Lyon, F-69008 France; Department of Preventive Medicine, Northwestern University Feinberg School of Medicine, 420 East Superior St, Chicago, IL 60611 USA; Veterans Affairs Boston Healthcare System, 150 South Huntington Ave, Boston, MA 02130 USA; Boston University School of Public Health, 715 Albany Street, Boston, MA 02118 USA; Instituto Nacional de Cancerología – Instituto de Investigaciones Biomédicas, Unidad de Investigación Biomédica en Cáncer, Universidad Nacional Autónoma de México, Mexico City, 14080 Mexico; Department of Biomedical Informatics, Faculty of Medicine, Universidad Nacional Autónoma de México, Mexico City, 04510 Mexico; Harvard School of Public Health, Landmark Center, Room 415E West, 401 Park Drive, PO Box 15677, Boston, MA 02215 USA

**Keywords:** *APOE*, *Epsilon*, Alleles, Haplotypes, Cognitive decline, Aging, Genetic variants

## Abstract

**Background:**

*APOE* is the biomarker with the greatest known influence on cognitive function; however, the effect of complex haplotypes involving polymorphisms rs449647, rs405509, rs440446, rs429358 and rs7412 has never been studied in older populations.

**Methods:**

We evaluated *APOE* polymorphisms using multiplex PCR for genotyping and Mini-Mental State Examination (MMSE) to evaluate cognitive function in 819 individuals from VA Normative Aging Study.

**Results:**

Combinatorial analysis of all polymorphisms and individual analysis of polymorphisms rs449647, rs405509, rs440446 and rs7412 did not show any association with cognitive performance. Polymorphism rs429358 was associated with better cognitive performance (odds of MMSE ≤ 25 = 0.63, 95% CI 0.42-0.95; *p* = 0.03) in the oldest subsample (5^th^ quintile of age) (odds of MMSE ≤ 25 = 0.34; 95% CI 0.13-0.86; *p* = 0.02). *APOE* allele ε4 was also associated with better cognitive performance (odds of MMSE ≤ 25 = 0.61, 95% CI 0.40-0.94; *p* = 0.02), also in the oldest subsample (odds of MMSE ≤ 25 = 0.35, 95% CI 0.14-0.90; *p* = 0.03).

**Conclusions:**

These results suggest a beneficial effect of polymorphism rs429358 in the oldest men.

**Electronic supplementary material:**

The online version of this article (doi:10.1186/s12888-014-0223-x) contains supplementary material, which is available to authorized users.

## Background

As the U.S. population ages, there is growing concern about the loss of mental acuity that is common through aging and associated with high financial cost [1], loss of independence [2] and mortality [3–5]. It has been estimated that at least 10% of people 65 years or older and 50% of those ≥ 85 years old have some form of cognitive impairment, ranging from mild deficits to dementia [6].

Biomarkers that predict future risks are critical to design targeted prevention of cognitive decline. Apolipoprotein E (APOE) is a protein involved in transport of cholesterol and lipids throughout the body. It also mediates clearance of plasma lipoproteins and contributes to redistribution of lipids to cells [7]. *APOE* ε4 allele, derived from the combination of polymorphisms rs429358 and rs7412, is the biomarker with the greatest known influence on the risk of developing Alzheimer’s disease (AD). Nonetheless, the ε4 allele has been found to be “protective” for AD in specific age groups, such as younger ages that precede the bulk of AD diagnosis [8–10] and in population older than 90 years old [11].

In addition to the single nucleotide polymorphisms (SNPs) used to define the *epsilon* alleles, two SNPs located in the promoter region of *APOE* have been described: rs449647 and rs405509 [12]. Evidence suggests that this region modulates transcriptional activity of *APOE* and that these polymorphisms may influence its effect on AD [13,14]. Two studies in populations from Italy have found an increased frequency of the A/A genotype and A allele of rs449647 in AD [15,16]. Bizarro et al. [15], and Lescai et al. [17], have reported higher frequencies of the G allele in rs405509 in AD than in controls (42.60% vs. 29.29%), whereas the frequency of the T allele for this polymorphism was lower in AD than in controls (32.5% vs. 48.0%) [15]. Polymorphism rs440446, located in intron 1 (enhancer), also affects transcriptional activity of *APOE* [18,19]. However, the effect of polymorphisms rs449647, rs405509, rs440446 and their haplotypes on cognitive function in older populations has never been studied. In this study, we evaluated the influence five *APOE* polymorphisms, including the *epsilon* alleles, in modulating cognitive function in men between the ages of 49 and 97 years participating in the prospective VA (Veterans Affairs) Normative Aging Study cohort.

## Methods

### Study participants

The U.S. Department of Veterans Affairs (VA) Normative Aging Study (NAS) is an ongoing longitudinal cohort that was established in 1963, which included men who were 21–81 years old and free of known chronic medical conditions at entry [20]. Men have been subsequently invited to medical examinations every three to five years. Additionally, participants completed cognitive testing since 1993. Participants who had experienced a stroke before the first cognitive test were excluded (3% of individuals), leaving a total of 819 individuals with cognitive testing and complete genotyping. The NAS study was approved by the Institutional Review Boards (IRB) at participating institutions and all participants provided written informed consent at each visit.

### Genotyping

Genetic polymorphism measurements included rs449647, rs405509, rs440446, rs429358 and rs7412. Multiplex PCR assays were designed using Sequenom SpectroDESIGNER software by inputting sequences containing the single nucleotide polymorphism (SNP) site and 100 bp flanking sequence on either side of the SNP. Most assays were genotyped using the Sequenom MassArray MALDI-TOF mass spectrometer (SpectroDESIGNER, Sequenom). Assays that failed to multiplex were genotyped using TaqMan 5′ exonuclease (Applied Biosystems, Foster City, CA) and ABI PRISM 7900 Sequence Detector System.

### Cognitive test

We administered the Mini-Mental State Examination (MMSE), a test of global cognition that assesses multiple cognitive areas, including orientation, immediate and short-term recall, attention and calculation, word finding, figure construction, reading and writing skills, and the ability to follow a 3-step command [21]. MMSE was designed as a dementia screening tool, but has been extensively validated and used in epidemiological research. The range of scores in MMSE is 0 to 30, corresponding to the lowest and the highest cognitive performance, respectively; however, in this study, the maximum MMSE score was 29 due to the exclusion of the question on the county of residence, which has limited meaning in Massachusetts [21,22]. We included cognitive data from study visits performed from 1993 through 2004. Although up to 4 cognitive tests were completed by some study participants, we analyzed only the first cognitive test for each participant to avoid bias related to better MMSE scores in repeated tests for each participant due to practice effect [23]. Also, we note that a large proportion of participants did not have repeated MMSE measures over time. Analyses considering cognitive decline are beyond the scope of this manuscript.

### Statistical analysis

Because of the presence of a ceiling effect at the highest MMSE score (MMSE score = 29), which was observed in 13.06% of our measures, we created a dichotomized variable for inferior MMSE performance. This approach has been used previously to provide more robust results on this dataset [24–26]”. As the MMSE is often used clinically to screen for dementia, inferior MMSE performance is likely to reflect clinically relevant cognitive impairment. Only 5.01% of our observations exhibited scores ≤24, which is the typical screening cut-off score used in research on dementia. Therefore we considered scores ≤25 as low performance (22.10% of our observations), as used in previous studies [27,28]. We evaluated the main effect of *APOE epsilon* alleles, complex haplotypes (observed combinations), and individual polymorphisms on the odds of having a low MMSE score using logistic regression with generalized estimating equations (GEE). All models were adjusted for potential confounders or predictors of cognitive function assessed at the time of the MMSE, including age at cognitive assessments (as a continuous variable), education (<12, 12–16, >16 years), total cholesterol (continuous), first language (English/not English), computer experience (yes/no), smoking (current, former, never), body mass index (BMI) (<25, ≥25 Kg/m^2^), physical activity (<12, 12–30, ≥30 metabolic equivalent hours (MET-hr) per week), alcohol intake (<2, ≥2 drinks/day), percentage of the participants that are non-white, percentage of the participants with at least a college degree, hypertension (yes/no), dark fish consumption (<once a week, ≥once a week), and diabetes (yes/no; defined as having reported diagnosis of diabetes or having fasting glucose above 126 mg/dl). To determine the presence of a beneficial effect in the youngest population, described as the “antagonistic pleiotropyc effect” and previously reported for *APOE* ε4 [29], a similar GEE approach was performed, stratified by quintiles of age. Additionally, to assess potential interaction between age and *APOE* ε4, we created a multiplicative term between age (as a continuous variable) and *APOE* ε4, and included it in the model along with the main effects. Hardy-Weinberg and linkage disequilibrium were determined using the PROC HAPLOTYPE statement from SAS software. Finally, all tests were two-sided and *p*-values < 0.05 were considered statistically significant. SAS software (Version 9.3, SAS Institute Inc., Cary, NC) was used for all statistical analyses.

## Results

### Characteristics of participants and frequencies

Most of the participants (48.72%) were in the 60–69 years age group, and 34.68% were 70–79 years old. All of the participants were male and 95% of them were white. The study sample had 7.33% current smokers and 50.67% reported 12–16 years of education (Table 1). The minimum value for years of education was 6 years. All five polymorphisms had minor allelic frequency of at least 7%, although the frequency of persons who were homozygous variant ranged from 0.5% to 23% (Table 2). We constructed a model of all the observed haplotypes using the three genetic conditions (wild-type, heterozygous, polymorphic homozygous) for each polymorphism and we found 54 combinations. The top ten most frequent haplotypes are shown in Table 3. Based on *epsilon* alleles distribution, alleles ε3, ε4 and ε2 showed frequencies of 78.91%, 13.87%, and 6.16%, respectively. Frequencies of *epsilon* haplotypes are also shown in Table 3.

**Table 1:** **Demographic characteristics of participants (n = 819)**

**Variable**	**n**	**%**
Age, years		
< 60	92	11.23%
60-69	399	48.72%
70-79	284	34.68%
≥80	44	5.37%
Education		
6-12 yrs	245	29.91%
12 to 16 yrs	415	50.67%
> 16 yrs	159	19.41%
Smoking status		
Never	245	29.91%
Former	514	62.76%
Current	60	7.33%
Alcohol consumption		
Yes	643	78.51%
No	176	21.49%
History of diabetes*		
Yes	719	87.79%
No	100	12.21%
Computer experience		
Yes	352	42.98%
No	467	57.02%
English as first language		
Yes	814	99.39%
No	5	0.61%
Hypertension		
Yes	504	61.54%
No	315	38.46%
BMI		
>25	640	78.14%
≤25	179	21.86%
Physical activity (MET-hr)		
<12	442	53.97%
12-30	217	26.50%
>30	160	19.54%
Dark fish consumption		
< once a week	674	82.30%
≥once a week	145	17.70%

*History of diabetes defined as having reported diagnosis of diabetes or having fasting glucose above 126 mg/dl.

MET-hr: metabolic equivalent hours.

**Table 2:** **Frequencies of genotypes and alleles in** ***APOE***

	**Genotypic frequencies**		**Allelic frequencies**	
rs449647	WT (AA)	64.47%	A	80.59%
	HT (AT)	32.23%	T	19.41%
	Poly (TT)	3.30%		
rs405509	WT (AA)	28.08%	A	52.63%
	HT (AC)	49.08%	C	47.37%
	Poly (CC)	22.83%		
rs440446	WT (GG)	40.78%	G	63.13%
	HT (GC)	44.69%	C	36.87%
	Poly (CC)	14.53%		
rs429358	WT (TT)	74.4%	T	86.20%
	HT (TC)	23.7%	C	13.80%
	Poly (CC)	2.0%		
rs7412	WT (CC)	85.59%	C	92.55%
	HT (CT)	13.92%	T	7.45%
	Poly (TT)	0.49%		

WT = Wild-type; HT = Heterozygous; Poly = Polymorphic.

**Table 3:** **Frequency of** ***APOE*** **haplotypes**

**Combinatorial**	**n**	**rs449647**	**rs405509**	**rs440446**	**rs429358**	**rs7412**	**Frequency**
a	125	AA	AC	GC	TT	CC	15.26%
b	109	AA	AA	GG	TT	CC	13.31%
c	97	AT	AC	GC	TT	CC	11.84%
d	68	AA	CC	CC	TT	CC	8.30%
e	52	AA	AC	GG	TC	CC	6.35%
f	35	AT	CC	CC	TT	CC	4.27%
g	31	AA	CC	GC	TC	CC	3.79%
h	27	AA	AA	GG	TC	CC	3.30%
i	27	AT	AA	GG	TT	CT	3.30%
j	21	AA	AA	GG	TT	CT	2.56%
Other	227						27.72%
***Epsilon***							
ε3ε2	93						11.36%
ε2ε2	4						0.49%
ε3ε4	174						21.25%
ε4ε4	20						2.44%
ε4ε1	15						1.83%
ε1ε1	1						0.12%
ε3ε3	512						62.52%

### Cognitive function according to APOE polymorphisms and haplotypes

**Figure 1:**
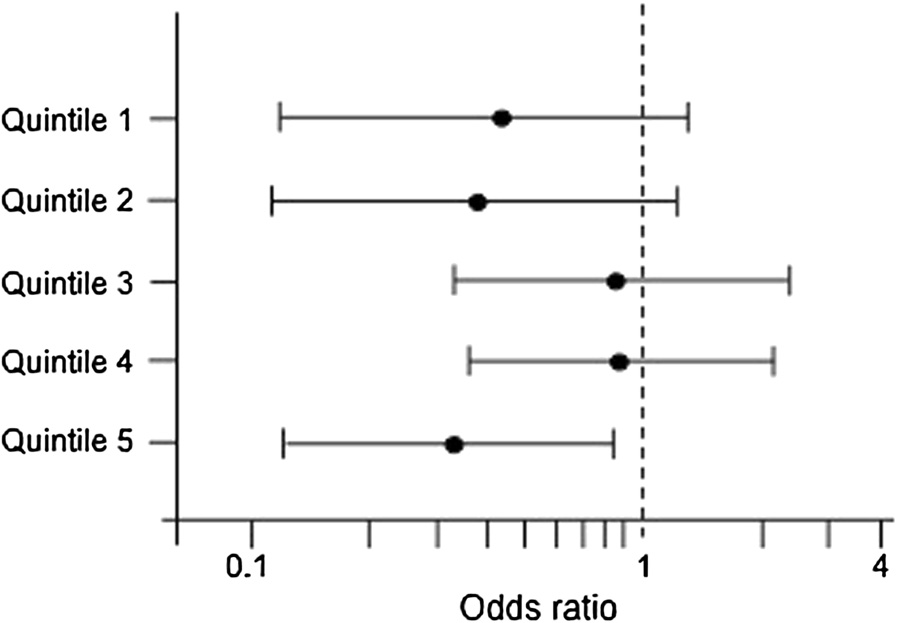
**Association between rs429358 polymorphism and MMSE ≤25 by quintiles of age.** Adjusted for race, education, alcohol consumption, physical activity, diabetes mellitus, fish consumption, computer experience, English as a first language, cholesterol, smoking, obesity, and hypertension. Results from wild type + heterozygous vs. polymorphic individuals. Q1-Q5 = 1^st^ to 5^th^ quintile. Numeric values for Figure 1 are shown in Additional file 1: Table S1.

Complex haplotypes (combination of genotypes) exhibited no association with MMSE ≤ 25 (*p* > 0.05) (Table 4). Also, analysis of each polymorphism was not associated with effect on cognitive performance for most of the alleles (Table 5). Only individuals homozygous for rs429358 minor allele exhibited better cognitive performance relative to wild type and heterozygous in both unadjusted (OR = 0.63, 95% CI 0.42-0.95 for the odds of MMSE ≤ 25; *p* = 0.03) and adjusted models (OR = 0.62, 95% CI 0.41-0.95; *p* = 0.03) (Table 5). Multivariable adjusted analysis of the effect of rs429358 on quintiles of age showed better cognitive performance only in the oldest population (5^th^ quintile of age, Q5) (OR for MMSE ≤ 25 = 0.34; 95% CI 0.13-0.86; *p* = 0.02) (Figure 1; Additional file 1: Table S1). Age also modified the association between rs429358 and cognitive function using the subset Q3-Q5 (OR using unadjusted model = 0.89, 95% CI = 0.80-0.99, *p* = 0.03; OR using fully adjusted model = 0.90, 95% CI 0.81-1.01; *p* = 0.072) (Additional file 1: Table S2).

**Table 4:** **Association between** ***APOE*** **combinatorial haplotypes and MMSE ≤25 (carriers vs. non-carriers)**

	**Unadjusted**		
**Combinatorial**	**OR**	**(95% CI)**	***p***
a	0.913	(0.572 - 1.457)	0.704
b	1.406	(0.890 - 2.221)	0.144
c	1.260	(0.773 - 2.053)	0.354
d	1.410	(0.807 - 2.462)	0.227
e	0.724	(0.346 - 1.514)	0.391
f	1.655	(0.795 - 3.447)	0.178
g	0.511	(0.177 - 1.481)	0.216
h	1.007	(0.400 - 2.534)	0.988
i	1.007	(0.400 - 2.535)	0.988
j	0.364	(0.084 - 1.578)	0.177

**Table 5:** **Association between** ***APOE*** **polymorphisms and MMSE ≤25**

		**Unadjusted**			Fully adjusted*		
	**Analysis**	**OR**	**(95% CI)**	***p***	**OR**	**(95% CI)**	***p***
rs449647	a	0.431	(0.128 - 1.449)	0.174	0.420	(0.121 - 1.462)	0.424
	b	1.190	(0.846 - 1.673)	0.317	1.166	(0.815 - 1.669)	0.401
rs405509	a	1.201	(0.819 - 1.762)	0.349	1.237	(0.824 - 1.857)	0.304
	b	0.994	(0.689 - 1.435)	0.975	0.955	(0.649 - 1.406)	0.817
rs440446	a	1.360	(0.873 - 2.120)	0.174	1.305	(0.813 - 2.094)	0.271
	b	1.024	(0.732 - 1.434)	0.889	0.997	(0.700 - 1.421)	0.989
rs429358	a	0.498	(0.112 - 2.212)	0.359	0.591	(0.125 - 2.799)	0.507
	b	0.634	(0.422 - 0.954)	**0.029**	0.620	(0.405 - 0.950)	**0.028**
rs7412	a	N/A			N/A		
	b	0.732	(0.442 - 1.211)	0.225	0.697	(0.411 - 1.182)	0.180

N/A: Low number of individuals.

OR = Odds Ratio; 95%CI = 95% Confidence Interval.

*Adjusted for race, education, alcohol consumption, physical activity, diabetes mellitus, fish consumption, computer experience, English as a first language, cholesterol, smoking, obesity, and hypertension.

Analysis a = (Wildtype + Heterozygous) vs. Polymorphic Homozygous genotypes. Analysis b = Wildtype vs. (Heterozygous + Polymorphic Homozygous) genotypes. Bold highlights statistical significant result.

**Figure 2:**
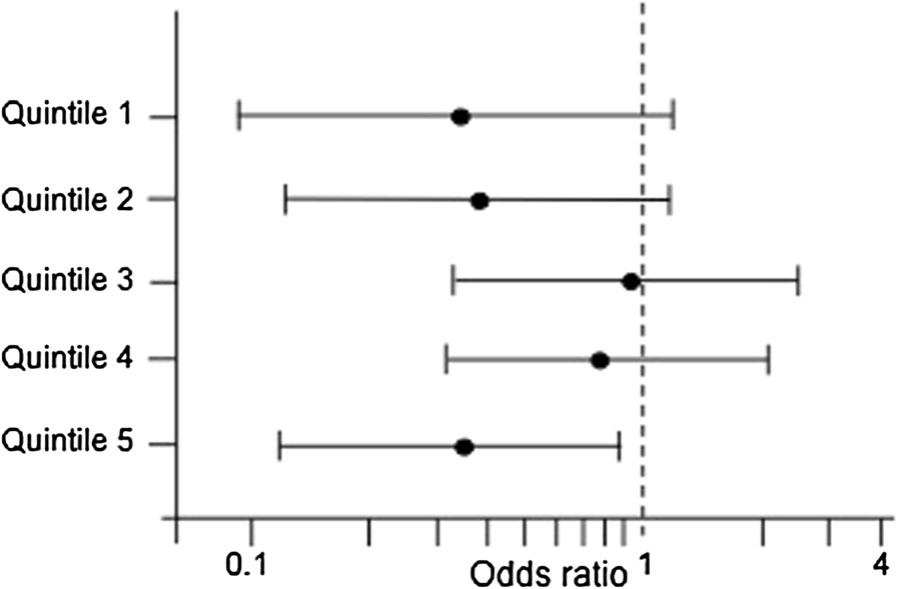
**Association between** ***APOE*** **ε4 status and MMSE ≤25 by quintiles of age.** OR = Odds ratio; Variability bars = 95% CI. Adjusted for race, education, alcohol consumption, physical activity, diabetes mellitus, fish consumption, computer experience, English as a first language, cholesterol, smoking, obesity, and hypertension. Results from carriers vs. non-carriers. Q1-Q5 = 1^st^ to 5^th^ quintile. Numeric values for Figure 1 are shown in Additional file 1: Table S3.

Analysis of *APOE* ε4 allele showed that this allele was also associated with better cognitive performance (odds of MMSE ≤25 = 0.61; 95% CI 0.40-0.94; *p* = 0.02) compared to non-carriers. Analysis by quintiles of age exhibited a progressive improvement of cognitive function associated with *APOE* ε4 in the population within the highest three quintiles of age (Q3-Q5), although *APOE* ε4 was significantly associated with better cognition only in the oldest group (Q5) (odds of MMSE ≤ 25 in Q5 = 0.35, 95% CI 0.14-0.90; *p* = 0.03) (Figure 2; Additional file 1: Table S3). Although all *APOE* haplotypes were tested, only ε4 decreased the risk of having low cognitive scores (Table 6). To determine the role of age in the association between *APOE* ε4 and cognitive function in the oldest group (Q5), we analyzed the effect of age using the last three quintiles of age (Q3-Q5). Results exhibited a modest interaction of age on the association between *APOE* ε4 and cognitive function in the oldest population (Q3-Q5) (OR using unadjusted model = 0.89, 95% CI = 0.81-0.99, *p* = 0.03; OR using fully adjusted model = 0.90, 95% CI 0.80-1.01; *p* = 0.08) (Additional file 1: Table S4). Additional analysis of polymorphisms rs449647, rs405509 and rs440446, adjusted by *APOE* ε4 allele, did not show modification of the risk.

**Table 6:** **Association between** ***APOE*** **epsilon alleles and MMSE ≤25 (carriers vs. non-carriers)**

	**Unadjusted**			Fully adjusted*		
**Allele**	**OR**	**(95% CI)**	***p***	**OR**	**(95% CI)**	***p***
ε1	0.498	(0.112 - 2.212)	0.359	0.615	(0.131 - 2.893)	0.538
ε2	0.781	(0.455 - 1.342)	0.371	0.784	(0.443 - 1.386)	0.402
ε2	2.043	(0.788 - 5.293)	0.141	2.103	(0.781 - 5.6603)	0.141
ε4	0.639	(0.425 - 0.961)	**0.032**	0.612	(0.398 - 0.940)	**0.024**

OR = Odds Ratio; 95%CI = 95% Confidence Interval.

*Adjusted for race, education, alcohol consumption, physical activity, diabetes mellitus, fish consumption, computer experience, English as a first language, cholesterol, smoking, obesity, and hypertension. Bold highlights statistical significant result.

All the polymorphisms analyzed were in Hardy-Weinberg equilibrium (Additional file 1: Table S5). Linkage disequilibrium analysis showed dependence between polymorphisms located at the 3′ *APOE* coding region, including the *APOE epsilon* constituents: rs424358 and rs7412 (D’ = 0.70). A strong linkage between other polymorphisms was also observed (Additional file 1: Table S6).

## Discussion

Our results showed that neither complex haplotypes – product of the combination of all *APOE* polymorphisms evaluated in the present study –, nor the polymorphisms rs449647, rs405509, rs440446, and rs7412 analyzed individually had any association with cognitive function. However, we found a protective effect of rs429358 on cognitive function, including a beneficial effect in the oldest population, as well as an interaction of age with the same polymorphism in determining cognitive function. Also, we found a protective effect of the *APOE* ε4 allele on cognition. Stratified analysis by quintiles of age also showed that *APOE* ε4 carriers exhibited better cognitive performance in the oldest subsample.

APOE is a protein associated with cholesterol-rich and triglyceride-rich plasma proteins. It is secreted into the circulation as a protein incorporated into very low-density lipoproteins (VLDLs), chylomicron remnants, and a subclass of high-density proteins. APOE regulates transport of cholesterol and lipids throughout the body and mediates clearance of plasma lipoproteins [30]. In the central nervous system, APOE is produced by astrocytes and circulates incorporated into small particles and disks resembling high-density lipoproteins. APOE contributes to redistribution of lipids to cells that require cholesterol and phospholipids [30].

*APOE* polymorphisms and haplotypes have been associated with AD and other human pathological conditions, but the effects of these combinations on healthy populations are not completely known. The frequencies of *APOE* genotypic and allelic frequencies analyzed here were similar to those reported in other samples [15,19,31]. Our results did not show associations between cognitive performance and any of *APOE* polymorphisms analyzed here, except for rs429358. This polymorphism exhibited a beneficial effect on cognitive function which was very similar to that observed for *APOE* ε4 allele (which is derived from the combination of polymorphisms rs429358 and rs7412), including its effect in the oldest population and the interaction of age between the polymorphism and cognitive function. This finding suggest that *APOE* ε4 effect could depend of rs429358 only, and is supported by the strong absence of linkage disequilibrium, a measurement of non-random association between alleles, between rs429358 and rs7412. Similar linkage disequilibrium between these two polymorphisms has been described previously [15]. On the other hand, although the main effect of the polymorphism could be influenced by the subset in the first two quintiles of age (Figure 1), the mediation analysis was statistically significant only using the oldest populations (Q3-Q5).

Recent work has analyzed cognitive and functional effects of the *APOE* ε4 allele. Evans et al. found than ε4+ individuals perform as well or even better than ε4- in middle age, possibly due to enhanced-age related frontal activity and diminished parietal recruitment [32]. These functional data indicate that *APOE* may participate in compensatory mechanisms at different life stages; these mechanisms could be activated in older individuals as a response to impaired activations of brain areas functionally affected during early ages, and could be reflected in better cognitive performance. Further studies are warranted to explore cognitive and neural functions in population of similar age as the NAS cohort.

Interestingly, we are not the first group reporting a better cognitive function in very old individuals carrying the *APOE* ε4 allele. Previous studies have found a reduction in the impact of the *APOE* ε4 allele on dementia in extreme old age, [33] and even a beneficial effect nonagenarians [11]; therefore, our findings in a subset of our population (the last quintile of age: mean = 77.8; min = 74, max = 97 years old) could reflect this previously reported effect. Additionally, some European studies have suggested that the effect of the APOE ε4 allele on dementia and mortality disappears in very old age [34]. Also, other studies have found that the relative prevalence of AD increased from the age of 60 to 89 years old and decreased slightly after age 90 [35]. On the other hand, beneficial effects of *APOE* ε4 allele have been reported in other human diseases, including myocardial infarction [36,37].

One of the factors that could influence our results is the role of sex hormones on the brain and on cognitive function, as it has been reported previously. AD affects approximately twice as many women as men and some studies have suggested that the effect of *APOE* ε4 on cognitive function is stronger in women than in men [8,9,38–42]. We recognize that gender/sex-hormones could interact in the association between genetic factors and cognitive performance, and therefore our result is applicable to men only.

Potential beneficial effects of *APOE* ε4 on cognition have been reported in children [41], teenagers [10], young adults [43], and middle-age adults [9,44]. Based on these findings, the *APOE* ε4 allele has been proposed as an example of antagonistic pleiotropic effect [45]. Antagonistic pleiotropy occurs when one gene controls for more than one trait, where at least one of these traits is beneficial to the organism’s fitness, and at least one is detrimental to the organism’s fitness [45]. However, our results did not show a beneficial effect of *APOE* ε4 allele in individuals less than 61 years old (1^st^ quintile of age, Q1). Previous additional studies have also failed in demonstrating the *APOE* antagonistic pleiotropic effect [46].

We recognize several limitations in our investigation. Our study is limited by using MMSE as the only score for cognitive function; therefore these results cannot be generalized to performance in specific cognitive domains, which needs to be evaluated in further analyses. Also, our findings are based on a cohort of older white men and may apply only to populations with similar characteristics. In fact, the impact of *APOE* ε4 allele has shown to vary in different populations, including an attenuated effect of *APOE* ε4 allele on Hispanics [47]. Tang et al., have also reported that *APOE* ε4 allele is a determinant in whites, but African Americans and Hispanics have an increased frequency of AD regardless of their APOE genotype [48]. On the other hand, a possible source of bias could be the inclusion of super-healthy people at the beginning of the study. Exploratory analysis deleting data from the subset of population older than 45 during the recruitment did not affected the results. Also, in this population, we did not collect data to investigate the role of cognitive reserve. Cognitive reserve may enhance cognition and modify the negative effects of pathological changes occurred during AD, as well as increase cognitive efficiency, capacity, and flexibility. Although our results were obtained after adjustment by education and computer experience, additional evaluations of specific domains, including literacy and vocabulary, may be better suited to reflect the effect of cognitive reserve [49].

We also recognize that the *APOE* ε4 allele has been consistently linked with impaired cognition in older populations [50–52]. We did not observe any negative effects of the APOE ε4 allele on cognitive function in our population. Several factors might explain this discrepancy, including gender, cognitive reserves and differential functional effect.

Additional studies are warranted to confirm our results among women and other ethnic groups. Although we attempted to control for a number of factors involved in cognitive function (e.g. age, education, computer experience, etc.), like any other epidemiological study, we cannot exclude the role of other potential confounders. Although our results suggest there is no effect of complex *APOE* haplotypes on cognitive function, we cannot conclude an absence of effect on cognition based on the low number of subjects in each group.

## Conclusions

Our study supports the possibility of a beneficial effect of rs429358 and *APOE* ε4 allele on cognition in subsets of the general population, specifically in the oldest men, and suggests that rs429358, and not the combination of other polymorphisms, may underlie the effect of the *APOE* ε4 allele.

## Additional file

Additional file 1: Table S1. Association between rs429358 polymorphism and MMSE ≤25 by quintiles of age. **Table S2.** Analysis of the interaction of age in the association between rs429358 and MMSE≤25. **Table S3.** Association between APOE ε4 status and MMSE ≤25 by quintiles of age. **Table S4.** Analysis of the interaction of age in the association between *APOE* allele ε4 and MMSE≤25. **Table S5.** Hardy-Weinberg analysis for each polymorphism. **Table S6.** Linkage disequilibrium analysis between *APOE* polymorphisms.

## Abbreviations

95% CI: 95% confidence interval
AD: Alzheimer’s disease
BMI: Body mass index
GEE: Generalized estimated equations
MET-hr: Metabolic equivalent hours
MMSE: Mini-mental state examination
NAS: Normative aging study
OR: Odds ratio
PCR: Polymerase chain reaction
Q1-Q5: 1^st^ to 5^th^ quintile
SNP: Single nucleotide polymorphism
U.S.: United States
VLDL: Very low-density lipoproteins
ε4: Epsilon 4
