# Supplementary material for: Influence of multiple APOE genetic variants on cognitive function in a cohort of older men – results from the Normative Aging Study
Source: BMC Psychiatry. 2014 Aug 1;14:223. doi: 10.1186/s12888-014-0223-x (PMC4149270; doi:10.1186/s12888-014-0223-x)
Supplement: Additional file 1: Table S1. — Association between rs429358 polymorphism and MMSE ≤25 by quintiles of age. Table S2. Analysis of the interaction of age in the association between rs429358 and MMSE≤25. Table S3. Association between APOE ε4 status and MMSE ≤25 by quintiles of age. Table S4. Analysis of the interaction of age in the association between APOE allele ε4 and MMSE≤25. Table S5. Hardy-Weinberg analysis for each polymorphism. Table S6. Linkage disequilibrium analysis between APOE polymorphisms. [file 12888_2014_223_MOESM1_ESM.docx]

SUPPLEMENTARY MATERIAL

| **Supplementary Table 1.** Association between rs429358 polymorphism and MMSE ≤25 by quintiles of age. | | | |  |
| --- | --- | --- | --- | --- |
| **Quintile of age** | **OR*** | **95% CI** | ***p*** | |
| Q1 (49-61 years) | 0.424 | (0.122 - 1.481) | 0.179 | |
| Q2 (62-65 years) | 0.399 | (0.122 - 1.308) | 0.129 | |
| Q3 (66-69 years) | 0.894 | (0.332 - 2.409) | 0.824 | |
| Q4 (70-73 years) | 0.909 | (0.366 - 2.261) | 0.838 | |
| Q5 (47-97 years) | 0.339 | (0.134 - 0.862) | **0.023** | |
| OR=Odds Ratio; 95%CI=95% Confidence Interval. | | | |  |
| *Adjusted for race, education, alcohol consumption, physical activity, diabetes mellitus, fish consumption, computer experience, English as a first language, cholesterol, smoking, obesity, and hypertension. | | | |  |
|  |  |  |  |  |
|  |  |  |  |  |

| **Supplementary Table 2.** Analysis of the interaction of age in the association between rs429358 and MMSE≤25. | | | | | | | | | | | | | | | | | | | | | | | | | | | |  |
| --- | --- | --- | --- | --- | --- | --- | --- | --- | --- | --- | --- | --- | --- | --- | --- | --- | --- | --- | --- | --- | --- | --- | --- | --- | --- | --- | --- | --- |
|  | | |  | |  | | |  | | | |  | | | |  | | |  | | |  | | | |  |  |  |
|  | | | **Unadjusted** | | | | | | | | | | | | | **Fully Adjusted*** | | | | | | | | | | | |  |
|  | | |  | | | |  | | | |  | |  | | |  | | | | |  | | | |  | |  | |
| **Parameter** | **OR** | | | **(95% CI)** | | | | | ***p*** | | | | | **OR** | | | **(95% CI)** | | | | | | ***p*** | | | | |  |
|  |  | | |  | |  | | |  | | | | |  | | |  | | |  | | |  | | | | |  |
| Intercept | 0.271 | (0.195-0.376) | | | | | | | | 0.034 | | | | | 0.168 | | | (0.039-0.728) | | | | | | 0.017 | | | |  |
| Age^§^ | 1.077 | (1.028-1.128) | | | | | | | | 0.002 | | | | | 1.082 | | | (1.026-1.140) | | | | | | 0.004 | | | |  |
| rs429358 | 1.230 | (0.634-2.385) | | | | | | | | 0.541 | | | | | 1.114 | | | (0.550-2.257) | | | | | | 0.764 | | | |  |
| Agexrs429358 | 0.894 | (0.805-0.992) | | | | | | | | **0.034** | | | | | 0.904 | | | (0.801-1.011) | | | | | | 0.078 | | | |  |
|  | | |  | | | |  | | | |  | |  | | |  | | | | |  | | | |  | |  | |
|  | | |  | | | |  | | | |  | |  | | |  | | | | |  | | | |  | |  | |
| OR=Odds Ratio; 95%CI=95% Confidence Interval.  *Adjusted for race, education, alcohol consumption, physical activity, diabetes mellitus, fish consumption, computer experience, English as a first language, cholesterol, smoking, obesity, and hypertension.  ^§^Age centered using age-(age median). | | | | | | | | | | | | | | | | | | | | | | | | | | | |  |
|  |  |  |  |  |  |  |  |  |  |  |  |  |  |  |  |  |  |  |  |  |  |  |  |  |  |  |  |  |
|  |  |  |  |  |  |  |  |  |  |  |  |  |  |  |  |  |  |  |  |  |  |  |  |  |  |  |  |  |

| **Supplementary Table 3.** Association between APOE ε4 status and MMSE ≤25 by quintiles of age. | | | |  |
| --- | --- | --- | --- | --- |
| **Quintile of age** | **OR*** | **(95% CI)** | ***p*** | |
| Q1 (49-61 years) | 0.355 | (0.094 - 1.341) | 0.127 | |
| Q2 (62-65 years) | 0.396 | (0.120 - 1.313) | 0.130 | |
| Q3 (66-69 years) | 0.935 | (0.330 - 2.647) | 0.899 | |
| Q4 (70-73 years) | 0.776 | (0.301 - 2.001) | 0.600 | |
| Q5 (47-97 years) | 0.351 | (0.138 - 0.895) | **0.028** | |
| OR=Odds Ratio; 95%CI=95% Confidence Interval.  *Adjusted for race, education, alcohol consumption, physical activity, diabetes mellitus, fish consumption, computer experience, English as a first language, cholesterol, smoking, obesity, and hypertension. | | | |  |

| **Supplementary Table 4.** Analysis of the interaction of age in the association between *APOE* allele ε4 and MMSE≤25. | | | | | | | | | | | | | | | |
| --- | --- | --- | --- | --- | --- | --- | --- | --- | --- | --- | --- | --- | --- | --- | --- |
|  |  |  |  |  | |  | |  | |  |  | | | | |
|  | **Unadjusted** | | | | | **Fully Adjusted*** | | | | | | | | | |
|  |  |  |  |  | |  | |  | |  |  | | | | |
| **Parameter** | **OR** | **(95% CI)** | | ***p*** | | **OR** | | **(95% CI)** | | | | ***p*** | |  |  |
|  |  |  |  |  | |  | |  | |  |  | | | | |
| Intercept | 0.269 | (0.194-0.374) | | | <0.0001 | | 0.168 | | (0.039-0.726) | | | | 0.017 | |  |
| Age^§^ | 1.078 | (1.029-1.129) | | | 0.002 | | 1.082 | | (1.027-1.141) | | | | 0.003 | |  |
| Allele ε4 | 1.264 | (0.650-2.457) | | | 0.489 | | 1.139 | | (0.562-2.311) | | | | 0.718 | |  |
| AgexAllele ε4 | 0.891 | (0.802-0.989) | | | **0.030** | | 0.902 | | (0.805-1.009) | | | | 0.072 | |  |
|  |  |  |  |  | |  | |  | |  |  | | | | |
|  |  |  |  |  | |  | |  | |  |  | | | | |

OR=Odds Ratio; 95%CI=95% Confidence Interval.

*Adjusted for race, education, alcohol consumption, physical activity, diabetes mellitus, fish consumption, computer experience, English as a first language, cholesterol, smoking, obesity, and hypertension.

^§^Age centered using age-(age median).

| **Supplementary Table 5.** Hardy-Weinberg analysis for each polymorphism. | | | | | | | |
| --- | --- | --- | --- | --- | --- | --- | --- |
|  |  |  |  |  |  |  |  |
| Polymorphism | Number of individuals | Number of alleles | Heterozygosity | Allelic Diversity | Test for HWE | DF | Pr > ChiSq |
| rs449647 | 819 | 2 | 0.322 | 0.313 | 0.746 | 1 | 0.388 |
| rs405509 | 819 | 2 | 0.491 | 0.499 | 0.199 | 1 | 0.655 |
| rs440446 | 819 | 2 | 0.447 | 0.447 | 1.315 | 1 | 0.251 |
| rs429358 | 819 | 2 | 0.237 | 0.238 | 0.014 | 1 | 0.904 |
| rs7412 | 819 | 2 | 0.139 | 0.138 | 0.076 | 1 | 0.783 |
|  |  |  |  |  |  |  |  |
|  |  |  |  |  |  |  |  |
| HWE=Hardy-Weinberg Equilibrium  DF=Degrees of Freedom | | |  |  |  |  |  |

| **Supplementary Table 6.** Linkage disequilibrium analysis between *APOE* polymorphisms. | | | | | | |
| --- | --- | --- | --- | --- | --- | --- |
|  |  |  |  |  |  |  |
|  |  | |  |  |  |  |
| D' | | | | | | |
|  | rs405509 | rs4404046 | | rs429358 | | rs7412 |
| rs449647 | 0.094 | 0.236 | | 0.721 | | 0.337 |
| rs405509 |  | 0.982 | | 0.306 | | 0.969 |
| rs4404046 |  |  | | 0.776 | | 0.946 |
| rs429358 |  |  | |  | | 0.702 |
|  |  |  | |  | |  |
|  |  |  | |  | |  |
| D'= Measure of linkage disequilibrium | | | | | |  |
